# Identification of 1,2,3,4,6-Penta-*O*-galloyl-β-d-glucopyranoside as a Glycine *N*-Methyltransferase Enhancer by High-Throughput Screening of Natural Products Inhibits Hepatocellular Carcinoma

**DOI:** 10.3390/ijms17050669

**Published:** 2016-05-04

**Authors:** Rajni Kant, Chia-Hung Yen, Chung-Kuang Lu, Ying-Chi Lin, Jih-Heng Li, Yi-Ming Arthur Chen

**Affiliations:** 1 Institute of Microbiology and Immunology, National Yang-Ming University, Taipei 11221, Taiwan; rajnihpmicro@gmail.com; 2 Center for Infectious Disease and Cancer Research (CICAR), Kaohsiung Medical University, Kaohsiung 80708, Taiwan; chyen@kmu.edu.tw; 3 Graduate Institute of Natural Products, College of Pharmacy, Kaohsiung Medical University, Kaohsiung 80708, Taiwan; 4 Lipid Science and Aging Research Center (CHY), Kaohsiung Medical University, Kaohsiung 80708, Taiwan; 5 Research Center for natural products and Drug Development (CHY), Kaohsiung Medical University, Kaohsiung 80708, Taiwan; 6 National Research Institute of Chinese Medicine, Taipei 11221, Taiwan; cklu@nricm.edu.tw; 7 Department of Life Sciences and Institute of Genome Sciences, College of Life Science, National Yang-Ming University, Taipei 11221, Taiwan; 8 School of Pharmacy, College of Pharmacy, Kaohsiung Medical University, Kaohsiung 80708, Taiwan; yclin@kmu.edu.tw (Y.-C.L.); jhlitox@kmu.edu.tw (J.-H.L.); 9 Ph.D. Program in Toxicology, College of Pharmacy, Kaohsiung Medical University, Kaohsiung 80708, Taiwan; 10 Institute of Biomedical Sciences, National Sun Yat-sen University, Kaohsiung 80708, Taiwan; 11 Department of Microbiology and Immunology, Institute of Medical Research and Institute of Clinical Medicine, College of Medicine, Kaohsiung Medical University, Kaohsiung 80708, Taiwan

**Keywords:** glycine *N*-methyltransferase (GNMT), hepatocellular carcinomas (HCC), 1,2,3,4,6-penta-*O*-galloyl-β-d-glucopyranoside (PGG), sorafenib, high-throughput screening (HTS)

## Abstract

Glycine *N*-methyltransferase (GNMT) expression is vastly downregulated in hepatocellular carcinomas (HCC). High rates of GNMT knockout mice developed HCC, while overexpression of GNMT prevented aflatoxin-induced carcinogenicity and inhibited liver cancer cell proliferation. Therefore, in this study, we aimed for the identification of a GNMT inducer for HCC therapy. We established a GNMT promoter-driven luciferase reporter assay as a drug screening platform. Screening of 324 pure compounds and 480 crude extracts from Chinese medicinal herbs resulted in the identification of *Paeonia lactiflora* Pall (PL) extract and the active component 1,2,3,4,6-penta-*O*-galloyl-β-d-glucopyranoside (PGG) as a GNMT inducer. Purified PL extract and PGG induced GNMT mRNA and protein expression in Huh7 human hepatoma cells and in xenograft tumors. PGG and PL extract had potent anti-HCC effects both *in vitro* and *in vivo*. Furthermore, PGG treatment induced apoptosis in Huh7 cells. Moreover, PGG treatment sensitized Huh7 cells to sorafenib treatment. Therefore, these results indicated that identifying a GNMT enhancer using the GNMT promoter-based assay might be a useful approach to find drugs for HCC. These data also suggested that PGG has therapeutic potential for the treatment of HCC.

## 1. Introduction

Globally, hepatocellular carcinoma (HCC) is one of the most prevalent malignancies and deadliest cancers [1,2]. To date, only sorafenib has been approved for the treatment of HCC [3,4]. However, it has side effects, and patients quickly develop resistance to it [5,6]. Therefore, there is an unmet need for more effective drugs for HCC [7].

Glycine *N*-methyltransferase (GNMT) possesses a tumor suppressive effect against HCC [8,9]. GNMT has protective effects against exposure to various carcinogens, including aflatoxins and polyaromatic hydrocarbons [10,11]. It is downregulated in the tumorous tissues of >80% HCC patients, as well as in the livers of cirrhotic patients who were at risk of developing HCC [12,13]. Furthermore, GNMT is downregulated in the liver tissues of nonalcoholic fatty liver (NFLD) patients and dietary rodent models of NFLD [14]. In addition, high rates of both genders of Gnmt knockout mice developed HCC spontaneously [8,15]. However, transgenic mice with human GNMT overexpression in their livers resisted aflatoxin B1-induced liver tumorigenesis [10]. Moreover, recent studies have shown that overexpression of GNMT inhibits cancer cell proliferation [16,17]. Therefore, we hypothesized that enhancement of GNMT expression would have a beneficial therapeutic effect on HCC.

Natural products have been regarded as an unlimited resource for drug discovery [18]. It has been estimated that up to 1990, nearly 80% of drugs were either natural products or their analogs [19]. Among natural products, traditional Chinese medicine has a unique position, since it represents a wide diversity of plants that has been used by human beings for a long period of time [20]. Here, we developed a GNMT promoter reporter assay and used it to screen a Chinese medicinal drug library. PGG was identified as a GNMT inducer from the extract of *Paeonia lactiflora* Pall (PL). We further characterized the inhibitory function of PGG on HCC both *in vitro* and *in vivo*.

## 2. Results

### 2.1. Establishment of Drug Screening Platform and Identification of Paeonia lactiflora Pall Extract as a Glycine N-Methyltransferase (GNMT) Inducer

**Figure 1:**
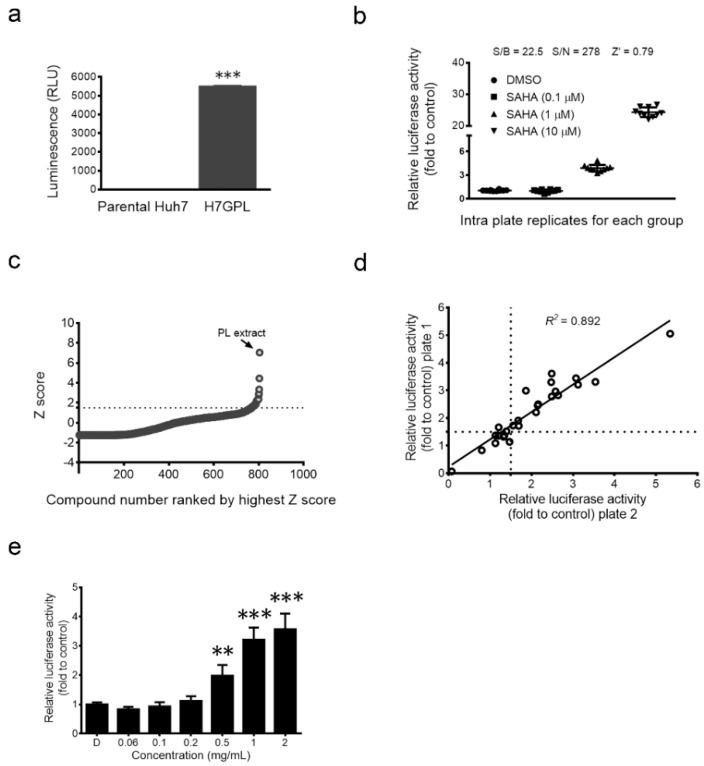
Establishment of a drug screening platform for high-throughput screening. (**a**) Huh7 GNMT promoter-luciferase (H7GPL) stable clone and parental human hepatoma Huh7 cells were harvested for luciferase activity measurement. Results are the means ± SD (*n* = 3); (**b**) Well-to-well reproducibility (*n* = 10) and dose response of Glycine *N*-methyltransferase (GNMT) promoter activity in H7GPL cells treated with Dimethyl sulfoxide (DMSO) or suberoylanilide hydroxamic acid (SAHA). Relative luciferase activity was calculated by normalizing luciferase activity to cell viability and presented as the fold to control after 24 h of drug treatment; (**c**) Primary screening plot shows the distribution of the *Z* score; the dashed line shows the cut off value (*Z* score > 1.5), and dots above this line represent hits; (**d**) XY-scatter plot of relative promoter activity illustrating the compounds that increased GNMT promoter reporter expression in the secondary screen. The cell viability was used to normalize the reporter activity and plotted as the fold to control. The “relative promoter activity Plate 1” and “Relative promoter activity Plate 2” represent the fold change for the two independent replicates of the screen. Dashed lines show the cut off value (fold change > 1.5), and dots above this line in both replicates were considered as hits; (**e**) H7GPL cells were treated with indicated concentrations of PL extract. After 24 h, the relative luciferase activity was determined as described above. The graph shows the means ± SD (*n* = 3). *** *p* < 0.001, ** *p* < 0.01 (Student’s *t*-test).

To identify the GNMT inducer, we established a GNMT promoter-driven firefly luciferase reporter stable cell line, the Huh7 GNMT promoter-luciferase (H7GPL) cell. Basal luciferase activity measured in H7GPL cells confirmed the presence of the reporter fragment (Figure 1a). We tested 26 drugs that can reverse the gene signatures of HCC [21] and found that 23% (6/26) of the tested drugs enhance GNMT promoter activity (Supplementary Table S1). Suberoylanilide hydroxamic acid (SAHA) was shown to be a potent GNMT inducer (Supplementary Table S1). We further evaluated the qualification of H7GPL cells as a drug screening platform with SAHA. H7GPL cells responded to SAHA dose-dependently and showed a small inter-well variation in a 96-well format (Figure 1b). Statistical parameters associated with the quality of the platform, such as signal-to-background (S/B) ratio (22.5), signal-to-noise (S/N) ratio (278) and *Z*’ factor (0.79), indicated that our platform met the acceptance criteria for high-throughput screening.

The library consisting of 324 pure compounds and 480 crude extracts from Chinese medicinal herbs was used for screening. The screening flowchart is described in the supplementary data (Supplementary Figure S1). We identified the extract of *Paeonia lactiflora* Pall (PL) as the highest inducer of GNMT promoter activity (Figure 1c–e).

### 2.2. The Anti-Hepatocellular Carcinomas (HCC) Effect of Paeonia lactiflora (PL) Extract-Derived Bioactive Fraction

**Figure 2:**
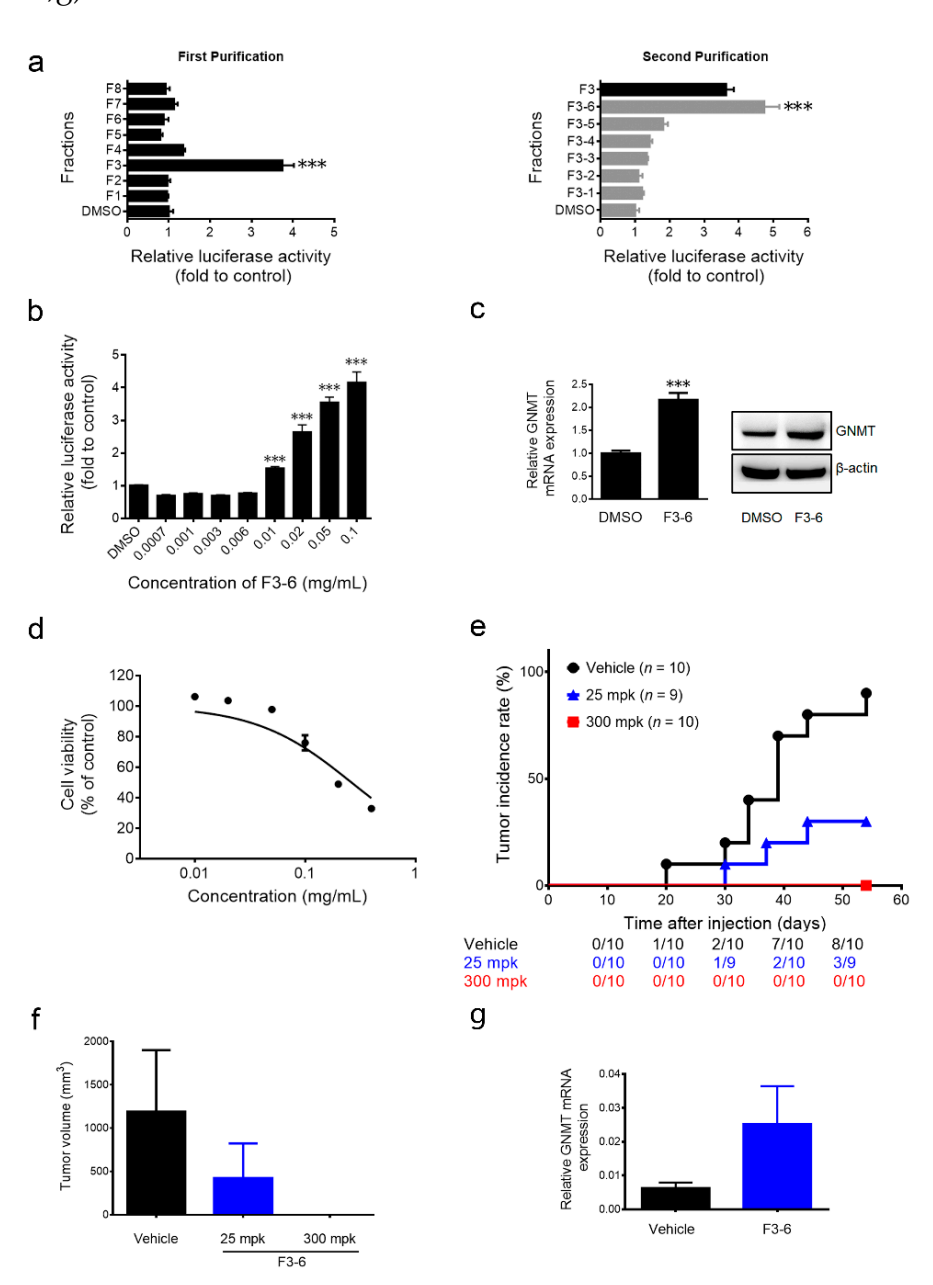
Partially-purified fraction Fraction F3-6 enhances GNMT gene expression and inhibits Huh7 cell growth. (**a**) Effect of PL extracts (0.1 mg/mL) and (**b**) indicated concentrations of F3-6 on GNMT promoter expression in H7GPL cells after 24 h of treatment. Relative luciferase activity was calculated and presented as mentioned in the Materials and Methods. Results are the means ± SD (*n* = 3); (**c**) Effect of F3-6 (0.1 mg/mL) on GNMT mRNA (left panel) and protein expression (right panel) in Huh7 cells after 24 h of treatment. Results are the means ± SD (*n* = 3); (**d**) The cell viability of Huh7 cells was analyzed after F3-6 treatment for 72 h, and cell viability was determined by the alamarBlue^®^ viability assay. The percentages of viable cells compared to the solvent control are plotted. Results are means ± SD (*n* = 3); (**e**) The overall tumor incidence curve of Huh7-implanted mice treated with F3-6 and vehicle control. The number of mice having a tumor at any given time point shown below; (**f**) Average tumor volume of each group at sacrifice. Columns, mean tumor volume for each group; bars, SE; (**g**) GNMT mRNA expression in tumors. The graph shows the means ± SE (*n* = 3). *** *p* < 0.001 (Student’s *t*-test).

Bioassay-guided fractionation was used to identify the active fractions and the effective compound in PL extract. Fraction F3-6 was the most potent fraction (Figure 2a) and induced GNMT promoter activity in a dose-dependent manner (Figure 2b). Moreover, F3-6 upregulated endogenous GNMT expression at mRNA and protein levels in Huh7 cells (Figure 2c). F3-6 inhibited the proliferation of Huh7 cells dose dependently (Figure 2d). Importantly, results from the xenograft assay showed that F3-6 delayed and reduced the incidence of tumor formation in mice (Figure 2e). The control group mice began to develop tumors at 20 days after they were inoculated with Huh7 cells, and 80% (8/10) of mice developed tumors within eight weeks. In contrast, it took 30 days for the first mice in the 25 mpk (mg per kg of body weight) F3-6-treated group to develop tumors, and the incidence reduced to 33% (3/9). Furthermore, no tumor was observed in mice treated with F3-6 at a dosage of 300 mpk during the experiment period (Figure 2e). Moreover, compared to the vehicle control group, the average size of the tumors in the F3-6 (25 mpk)-treated group decreased remarkably with an elevated mRNA level of GNMT (Figure 2f,g).

### 2.3. 1,2,3,4,6-Penta-O-galloyl-β-d-glucopyranoside (PGG), the Active Component in F3-6, Inhibits Liver Cancer Cells’ Growth both in Vitro and in Vivo

**Figure 3:**
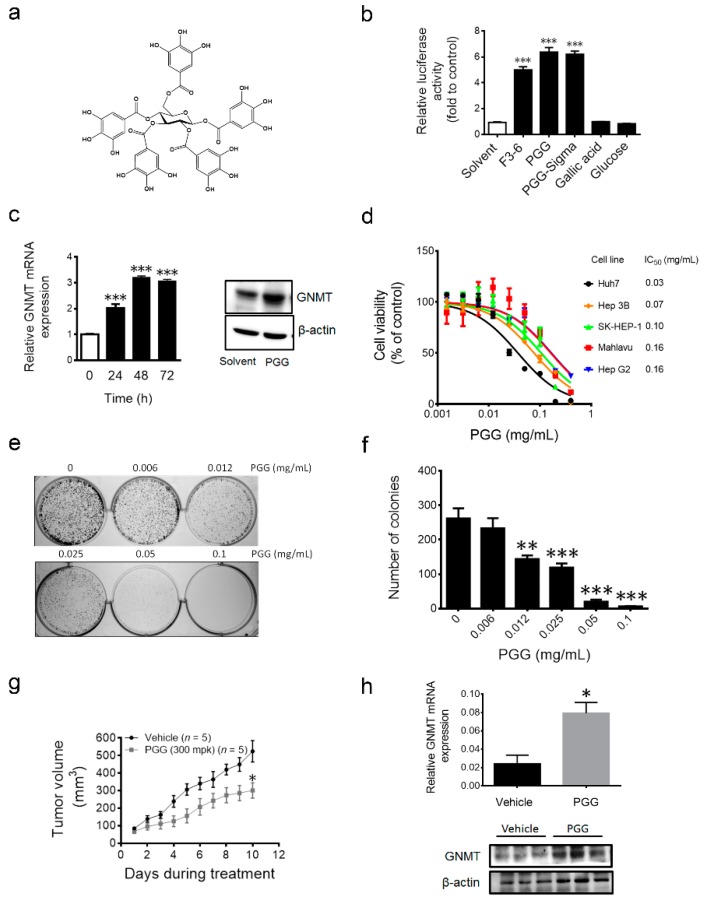
Effect of PGG treatment on liver cancer cells *in vitro* and *in vivo*. (**a**) Chemical structure of PGG; (**b**) Effect of indicated compounds (0.1 mg/mL) on GNMT promoter expression in H7GPL cells after 24 h of treatment. Data are presented as the fold to solvent control. Results are the means ± SD (*n* = 3); (**c**) Endogenous GNMT mRNA (left panel) and protein level (right panel) in PGG (0.1 mg/mL)-treated Huh7 cells. The graph shows the means ± SD (*n* = 3); (**d**) Dose response viability curve of indicated liver cancer cell lines treated with PGG for 72 h. The percentages of viable cells compared to the solvent control are plotted. IC_50_ values of PGG in each cell line are shown. Results are the means ± SD (*n* = 3); (**e**) Long-term effect of PGG on Huh7 growth was determined by the colony formation assay (representative image); (**f**) The graph shows the quantification of colony numbers in (**e**). Results are the means ± SD (*n* = 3); (**g**) Growth curves of Huh7 xenograft tumors in mice treated with PGG and vehicle control. Error bars represent the SE (*n* = 5); (**h**) GNMT mRNA (upper panel) and protein expression in tumor samples (lower panel) SE (*n* = 3). *** *p* < 0.001, ** *p* < 0.01, * *p* < 0.05 (Student’s *t*-test).

The F3-6 fraction was further purified by HPLC and 1,2,3,4,6-penta-*O*-galloyl-β-d-glucopyranoside (PGG, purity >98%) was identified as the active component in fraction F3-6 (Figure 3a and Supplementary Figure S2). PGG is a hydrolysable tannin composed of glucose and gallic acid. As shown in Figure 3b, PGG either purified from F3-6 or purchased from Sigma induced GNMT promoter activity, while neither glucose nor gallic acid showed similar effects as PGG. Furthermore, PGG induced GNMT mRNA, as well as protein expression in Huh7 cells (Figure 3c). Next, we analyzed the effect of PGG on cell proliferation in different liver cancer cell lines, including Huh7, Hep 3B, SK-HEP-1, Mahlavu and Hep G2. Results showed that PGG inhibited the proliferation of all tested liver cancer cells in a dose-dependent manner (Figure 3d). Furthermore, PGG reduced the colony formation of Huh7 cells in a dose-dependent manner (Figure 3e,f). Finally, a significant suppression of xenograft tumor growth was observed in the PGG-treated mice compared to vehicle control (Figure 3g). Finally, qPCR and immunoblot analysis of tumor samples revealed that GNMT was induced in the PGG-treated group (Figure 3h).

### 2.4. PGG Treatment Induces Apoptosis in Huh7 Cells

**Figure 4:**
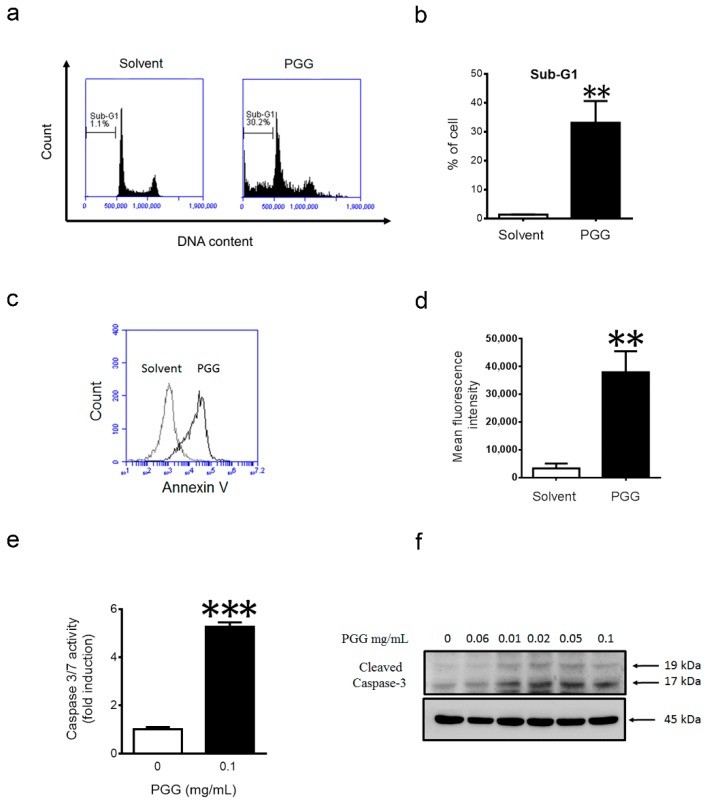
PGG treatment induces apoptosis in Huh7 cells. (**a**) Huh7 cells were harvested for PI staining and flow cytometry analysis after 24 h of PGG (0.1 mg/mL) treatment; (**b**) The quantification of the sub-G1 population in (**a**). Results are the means ± SD (*n* = 3); (**c**) Huh7 cells were harvested for Annexin V staining and flow cytometry analysis after 12 h of PGG treatment (0.1 mg/mL); (**d**) The quantification of fluorescence intensity in (**c**). Results are the means ± SD (*n* = 3); (**e**) Huh7 cells were harvested for caspase 3/7 assay after 12 h of PGG (0.1 mg/mL) treatment. Caspase 3/7 activity were expressed as the fold to solvent control. Results are the means ± SD (*n* = 3); (**f**) Immunoblot analysis showing induction of cleaved caspase 3 after 12 h of treatment with indicated concentrations of PGG. β-actin expression was used as the loading control. *** *p* < 0.001, ** *p* < 0.01 (Student’s *t*-test).

Next, we explored the mechanism by which PGG inhibits liver cancer cell proliferation. Treatment of Huh7 cells with PGG for 24 h resulted in significant increases in the sub-G1 population (Figure 4a,b), as well as Annexin-V-positive cells (Figure 4c,d), indicating that PGG could induce apoptosis. Consistent with these findings, a five-fold increase in caspase 3/7 activity was observed in Huh7 cells after 12 h of PGG treatment (Figure 4e). Immunoblot further confirmed that PGG treatment increased the level of cleaved caspase 3 in a dose-dependent manner (Figure 4f). Taken together, PGG caused apoptosis in Huh7 cells.

### 2.5. PGG Sensitizes Huh7 Cells to Sorafenib Treatment

**Figure 5:**
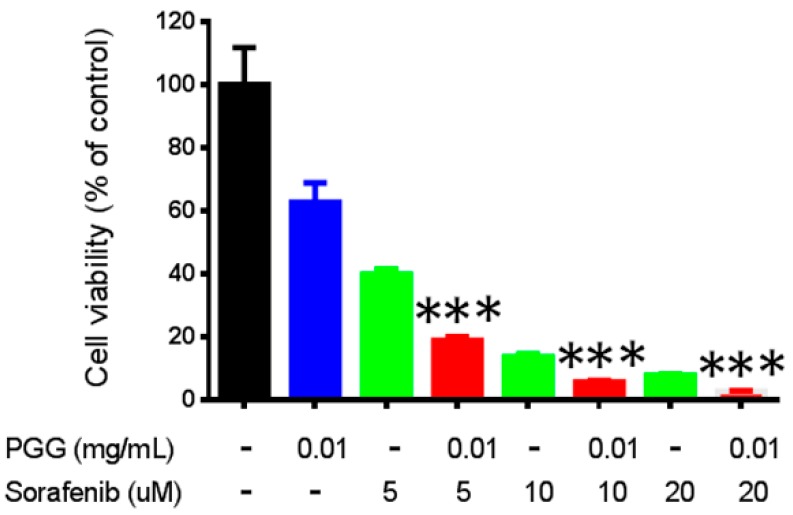
PGG sensitizes Huh7 cells to sorafenib treatment. Huh7 cells were treated with indicated concentrations of PGG (blue), sorafenib (green) or combinations (red) for 72 h. Cell viability was measured using the alamarBlue^®^ assay. The percentages of viable cells compared to the solvent control are plotted. Results are the means ± SD (*n* = 3). *** *p* < 0.001 (Student’s *t*-test).

To date, sorafenib is considered the drug of choice for HCC, but its effectiveness is limited, and its side effects are still a major concern [6]. Moreover, ongoing studies are seeking to increase the efficacy of sorafenib by combining it with other drugs or therapeutic interventions. Therefore, we investigated whether PGG treatment can sensitize hepatoma cells to sorafenib treatment. As shown in Figure 5, co-exposure with PGG significantly enhanced the effect of sorafenib on the viability of Huh7 cells.

## 3. Discussion

GNMT has a tumor suppressive role in HCC [9]. It is highly abundant in normal hepatocytes, but is barely detectable in HCC [22]. It has been shown that restoration of GNMT in HCC cell lines results in repression of cell proliferation and tumor growth [16]. In this study, we developed a GNMT-oriented drug screening platform to identify potential drug candidates for HCC. We tested 26 drugs previously reported to reverse the gene expression signature of HCC [21]. Six compounds (23%) were identified as capable of increasing GNMT promoter activity. Most of them have never been reported as enhancers of GNMT. We reasoned that our GNMT-based approach could be useful in discovering potential drugs for HCC. Subsequently, we used such an assay to screen a traditional Chinese medicine drug library and identified PGG from the extracts of *Paeonia lactiflora* Pall having an anti-HCC effect *in vitro* and *in vivo*.

The *Paeonia lactiflora* Pall root, a famous traditional Chinese medicine, has been used for the treatment of hepatic diseases for years [23]. Here, we showed that PGG purified from *Paeonia lactiflora* Pall sensitizes Huh7 cells to sorafenib treatment. Sorafenib is the only oral-administrated drug approved for advanced HCC, while concerns, including the efficacy, adverse effects and drug resistance, have been raised [5,6]. The combination of herbal medicines or natural products with conventional anti-cancer agents has been shown to produce beneficial effects [24]. Thus, it is worthy to further evaluate the efficacy of sorafenib in combination with PL extract or PGG for HCC treatment in more models or clinically.

PGG is a natural product that exhibits multiple pharmacological activities, including anti-cancer effects on different types of cancers [25]. Oh *et al.* [26] showed that PGG induces G0/G1 phase arrest and inhibits NF-κB in SK-HEP-1 cells. Yin *et al.* [27] demonstrated that PGG induces senescence-like S-phase arrest in hepatoma and breast cancer cells. Dong *et al.* [28] reported that PGG induces autophagy-mediated senescence-like arrest in liver, breast and lung cancer cells. In the present study, PGG was found to be a GNMT gene expression inducer for the first time. The mechanism of GNMT promoter induction by PGG needs further study.

Several reports on the safety of PGG have been published [25]. We tested the safety of PGG supplementation in C57BL/6 mice for 28 days. PGG was well tolerated; no significant weight loss and organ damage were observed in tested mice (Supplementary Figure S3). Moreover, the Ames test showed that PGG was not mutagenic both in the presence and absence of metabolic activation (Supplementary Tables S2–S4). Furthermore, its anti-mutagenic activity was reported previously. All of these results supported the safety of PGG in clinical use as a single treatment or adjuvant therapy.

GNMT is highly downregulated in HCC, and the downregulation mechanism is still not clear [9]. Huidobro *et al.* [29] have reported that DNA methylation contributes to the gene repression of GNMT in HCC. However, in their report, the demethylating agent (AdC) did not show notable induction in GNMT mRNA expression in HCC cells (<1.5-fold induction). Histone deacetylation is known as one of the mechanisms for the inactivation of tumor suppressor genes in cancer [30]. Our results showed that histone deacetylase inhibitors SAHA, as well as trichostatin A (TSA) remarkably induced GNMT promoter activity [31]. Moreover, we found a three-fold induction in GNMT mRNA expression in TSA-treated Huh7 cells (Supplementary Figure S4). For the first time, our data related histone deacetylation to inactivation of GNMT in HCC. Moreover, identifying PGG’s enhancement mechanism might help us to understand the pathways involved in GNMT downregulations. The possible mechanism underlying GNMT induction by PGG is under investigation.

In summary, we established and evaluated a GNMT promoter-driven drug screening assay as a valuable platform for high throughput screening of compounds for the treatment of HCC. Importantly, we revealed a novel bioactivity of PGG, which has been reported to possess anti-HCC properties, as a GNMT enhancer. Results suggested that GNMT induction by PGG treatment may play an important role in the anti-HCC activities of PGG. Thus, studies of PGG and its effect on GNMT expression in HCC will require further investigation. Furthermore, our result is the first, to our knowledge, to report that the combination of PGG with sorafenib inhibited Huh7 cell growth to a greater extent than either component alone. These data indicate a potential application of a novel combination in the future treatment of HCC. Moreover, this study, for the first time, linked histone deacetylation to GNMT promoter activity and provided important insight for understanding the mechanism of GNMT downregulation in HCC.

## 4. Materials and Methods

### 4.1. Cell Culture and Reagents

Human liver cancer cell lines Huh7, Hep G2, Hep 3B, SK-HEP-1, Mahlavu and embryonic kidney cell line HEK-293T all were cultured in Dulbecco’s Modified Eagle’s Medium (DMEM) (Gibco BRL, Grand Island, NY, USA) with 10% heat-inactivated fetal bovine serum (HyClone, Logan, UT, USA), penicillin (100 U/mL), streptomycin (100 μg/mL), nonessential amino acids (0.1 mM/L) and l-glutamine (2 mM/L) in a humidified incubator with 5% CO_2_. Stable cells established by lentiviral-system H7GPL were grown in DMEM supplemented with 1 μg/mL puromycin.

### 4.2. Plasmids and Transfections

Plasmids for lentivirus production (pCMV-ΔR8.91, pMD.G and pLKO.1) were obtained from the National RNAi Core Facility (Academia Sinica, Taipei, Taiwan). The −1812/+14 GNMT promoter-luciferase plasmid was constructed in our previous study [32]. For transfection, cells were plated to 70%–90% confluence, and plasmid DNAs were transfected by using TurboFect Reagent (Fermentas, Hanover, MD, USA). All transfections were performed according to the manufacturer recommendation.

### 4.3. Development of GNMT Expression-Oriented Drug Screening Platform

The plasmid-pLKO.1 was purchased from RNAi Core (Academia Sinica). The U6 promoter fragment located within the ClaI and EcoRI site of pLKO.1 was replaced by a ClaI-BamHI-EcoRI (CBE) linker. The CBE linker was generated by annealing two oligonucleotides: 5′-CGATATCGGATCCGTCGACG-3′ and 5′-AATTCGTCGACGGATCCGATAT-3′. The resultant double-stranded DNA fragment contained a 5′ overhang end of ClaI followed by an EcoRV site, a BamHI site, a SalI site and a 5′ overhang end of EcoRI. The CBE linker was ligated to ClaI and EcoRI digested pLKO.1 vector to generate pLV-CBE plasmid. A ~4 kilobase (kb) fragment containing a synthetic poly(A) signal element (from the pGL3 promoter vector (Promega, Madison, WI, USA)), the GNMT promoter, the firefly luciferase reporter gene and Simian vacuolating virus 40 (SV40) late poly(A) signal elements were generated by digesting −1812/+14 GNMT promoter-luciferase plasmid with SspI and SalI. The resulting fragment was ligated into EcoRV and SalI digested pLV-CBE plasmid. The resultant plasmid was designated as pLV-GNMTpLuc and was used for lentivirus production. In brief, HEK293T cells were co-transfected with a packaging plasmid-pCMV-ΔR8.91, a VSV-G envelope-expressing plasmid-pMD.G and pLV-GNMTpLuc using TurboFect™ Reagent (Fermentas). Supernatant containing lentiviruses was harvested according to the protocol published on the website (http://rnai.genmed.sinica.edu.tw/). Huh7 cells were infected with this virus and selected with puromycin-containing medium. One resultant colony was picked, amplified and denominated as H7GPL (Huh7 GNMT promoter-luciferase). The cell lysate of H7GPL was used for luciferase activity measurement using the Luciferase Assay System (Promega). To determine the quality of the platform for drug screening, the promoter activities were used to calculate the statistical parameters, such as the signal-to-background (S/B) ratio, the signal-to-noise (S/N) ratio and the value of the *Z*’ factor according to the equations published by Zhang *et al.* [33]. H7GPL cells were used to screen the traditional Chinese medicinal drug library from the National Research Institute of Chinese Medicine as detailed in the supplementary methods.

### 4.4. Quantitative Real-Time PCR (qRT-PCR)

RNA was prepared by using Tri Reagent (Sigma-Aldrich, St Louis, MO, USA) and was reverse transcribed into cDNA using a Super Script II Reverse Transcriptase Kit (Invitrogen Inc., Carlsbad, CA, USA). PCR was performed on an ABI StepOne Plus System (Applied Biosystems, Foster City, CA, USA) using the LightCycler^®^ First Start DNA Master SYBR Green I reagent (Roche Diagnostics, Basel, Switzerland). The mRNA level was normalized using the TATA-box binding protein (TBP) mRNA level as the standard. The following primers were used: GNMT forward, 5′-ACTGGATGACTCTGGACAA-3′ and reverse, 5′-ACTGAGGATGTGGTCGT-3′; TBP forward, 5′-TGCACAGGAGCCAAGAGTGAA-3′ and reverse, 5′-CACATCACAGCTCCCCACCA-3′.

### 4.5. Immunoblotting

Cells or xenograft tumors were harvested with RIPA lysis buffer (50 mM Tris (pH 7.5), 150 mM NaCl, 1% Triton X-100, 0.1% sodium dodecyl sulfate (SDS), 0.5% sodium deoxycholate, supplemented with protease and phosphatase inhibitors (1 mM Phenylmethanesulfonyl Fluoride (PMSF), 10 μg/mL leupeptin, 50 μg/mL Tosyllysine Chloromethyl Ketone (TLCK), 50 μg/mL Tosyl phenylalanyl chloromethyl ketone (TPCK), 1 μg/mL aprotinin, 1 mM sodium fluoride (NaF), 5 mM sodium pyrophosphate (NaPPi) and 10 mM sodium orthovanadate (Na_3_VO_4_). Protein lysates were quantified and separated on SDS–PAGE gel, and immunoblotting was carried out as described previously [16]. The following antibodies were used: anti-GNMT (14-1, YMAC Bio Tech, Taiwan), anti-caspase-3 (9H19L2, Thermo Scientific) and anti-β-actin (AC-15, Sigma-Aldrich).

### 4.6. Cell Viability and Colony Formation Assay

AlamarBlue^®^ assay (AbD Serotec, Raleigh, NC, USA) was used to evaluate the cytotoxic effects of the tested drugs according to the manufacturer’s recommendation. For the colony assay, Huh7 cells were seeded (1 × 10^4^ cells/well) in 6-well plates. After an overnight incubation, cells were treated with drugs at the specified concentrations for 7 days. Numbers of surviving colonies were analyzed through the crystal violet staining method [34]. Colonies were quantified by OpenCFU colony counting software (http://opencfu.sourceforge).

### 4.7. Flow Cytometry

Cells were harvested and fixed with cold 75% ethanol overnight and stained with propidium iodide (10 μg/mL) and RNase A (1 mg/mL) at 37 °C for 30 min. The cells were then analyzed by an Accuri C6 Flow cytometer (BD Biosciences, San Jose, CA, USA). Cells were stained for Annexin V according to the manufacturer’s protocol (eBioscience, San Diego, CA, USA) and analyzed by an Accuri C6 Flow cytometer (BD Biosciences).

### 4.8. Caspase 3/7 Activity Assay

Huh7 cells were plated in 96-well plate and treated with solvent or PGG for 12 h. To measure caspase-3 and caspase-7 activation, the Apo-ONE^®^ Homogeneous Caspase-3/7 assay was performed according to the Promega Technical Bulletin-Apo-ONE^®^ Homogeneous Caspase-3/7 assay.

### 4.9. In Vivo Tumor Models

All animal experiments were reviewed and approved by the Institutional Animal Care and Use Committee of Kaohsiung Medical University (city, country) and performed in accordance with relevant guidelines. For the tumor incidence assay, 5–6-week-old female nonobese diabetic/severe combined immunodeficiency (NOD-SCID) mice were subcutaneously injected with Huh7 cells (1 × 10^6^) in the right flank. Five days after transplantation, mice were randomly divided into 3 groups and were treated with F3-6 (25 mg/kg (mpk) by i.p injection and 300 mpk by oral administration) and vehicle three times per week for 53 days. To test the antitumor effect of PGG *in vivo*, Huh7 cells (2 × 10^6^) were implanted subcutaneously into Balb/c nude mice as described above. Upon detection of palpable tumor, mice were randomly grouped and treated with PGG and drug vehicle orally every day for 10 days. Tumor growth was monitored (every day or three times per week) by using Vernier caliper measurement of the length (L) and width (W) of the tumor. Tumor volume (TV) was calculated by using the formula volume = (L × W^2^)/2. F3-6 was dissolved in PBS, PGG was dissolved in PBS or 0.5% carboxymethyl cellulose sodium salt.

### 4.10. Statistical Analysis

Statistical analyses were performed using MS excel and GraphPad Prism 5.0 software (La Jolla, CA, USA). Cumulative tumor incidence curves were analyzed using the Kaplan–Meier method. *p*-values were calculated using the unpaired two-sided Student’s *t*-test to compare groups, and *p* < 0.05 was considered statistically significant.

## Supplementary Materials

Supplementary materials can be found at http://www.mdpi.com/1422-0067/17/5/669/s1.

## Abbreviations

GNMT	glycine *N*-methyltransferase
HCC	hepatocellular carcinomas
PL	*Paeonia lactiflora* Pall
PGG	1,2,3,4,6-penta-*O*-galloyl-β-d-glucopyranoside
NFLD	nonalcoholic fatty liver
qPCR	quantitative real-time PCR
SAHA	suberoylanilide hydroxamic acid
TSA	trichostatin A
S/B	signal-to-background
S/N	signal-to-noise
mpk	mg per kg of body weight
