# Supplementary material for: Identification of 1,2,3,4,6-Penta-O-galloyl-β-d-glucopyranoside as a Glycine N-Methyltransferase Enhancer by High-Throughput Screening of Natural Products Inhibits Hepatocellular Carcinoma
Source: Int J Mol Sci. 2016 May 4;17(5):669. doi: 10.3390/ijms17050669 (PMC4881495; doi:10.3390/ijms17050669)
Supplement: Supplementary file 1 [file ijms-17-00669-s001.pdf]

Supplementary Materials: Identification of 1,2,3,4,6-Penta-*O*-galloyl-β-D-glucopyranoside as a Glycine *N*-Methyltransferase Enhancer by High-Throughput Screening of Natural Products Inhibits Hepatocellular Carcinoma

Rajni Kant, Chia-Hung Yen, Chung-Kuang Lu, Ying-Chi Lin, Jih-Heng Li and Yi-Ming Arthur Chen

1. Supplementary Figures and Legends

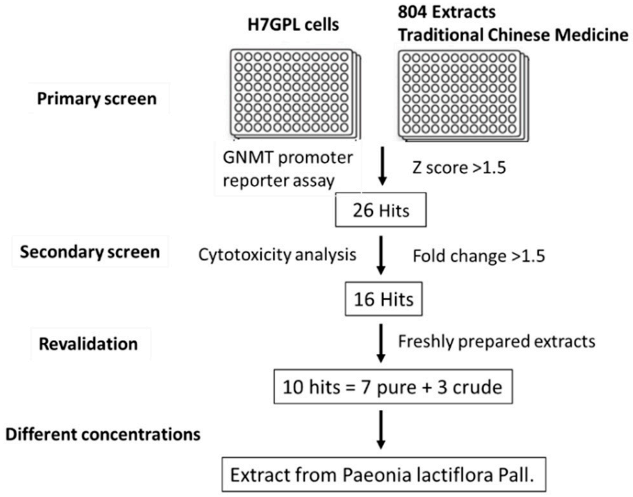

Figure S1. Flowchart of high-throughput screening. We used H7GPL cells to screen a traditional Chinese medicine drug library consisting of 324 pure compounds and 480 crude extracts from Chinese medicinal herbs. Hits of primary screening were sorted by Z score ≥ 1.5. There were 26 hits (13 pure compounds and 13 herb extracts) obtained and used for secondary screening. Hits of secondary screening were sorted by reporter activity (≥1.5-folds compared to DMSO solvent control), and 16 hits were identified and used for revalidation. After revalidation, 10 hits remained positive and tested for dose dependency. Among them, the extract from *Paeonia lactiflora* Pall (PL extract) was found to induce the highest increase in reporter activity and was chosen for further characterization.

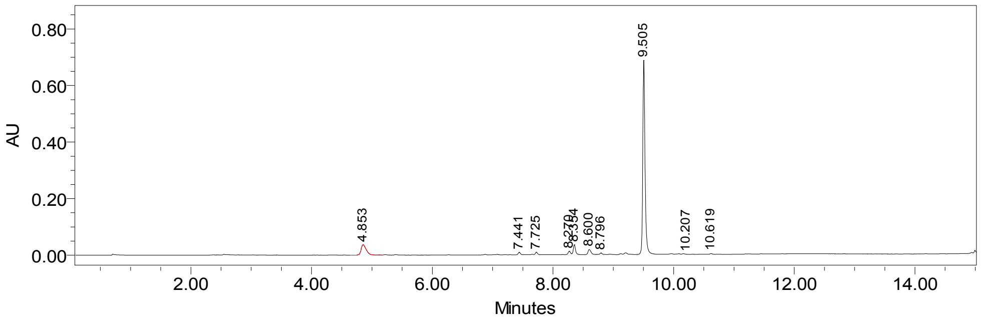

(a)

Figure S2. *Cont.*

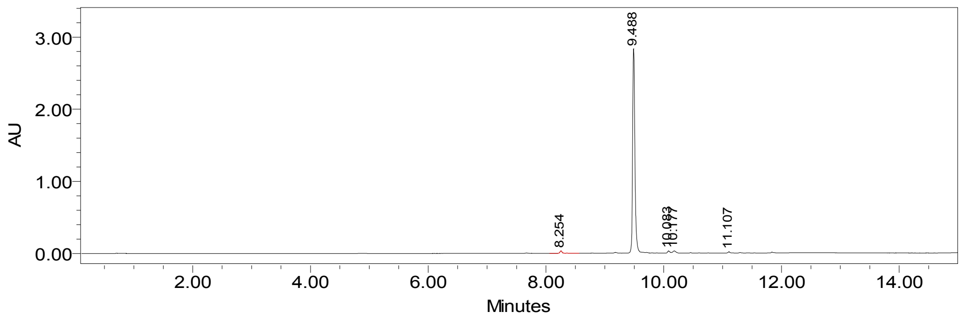

(**b**)

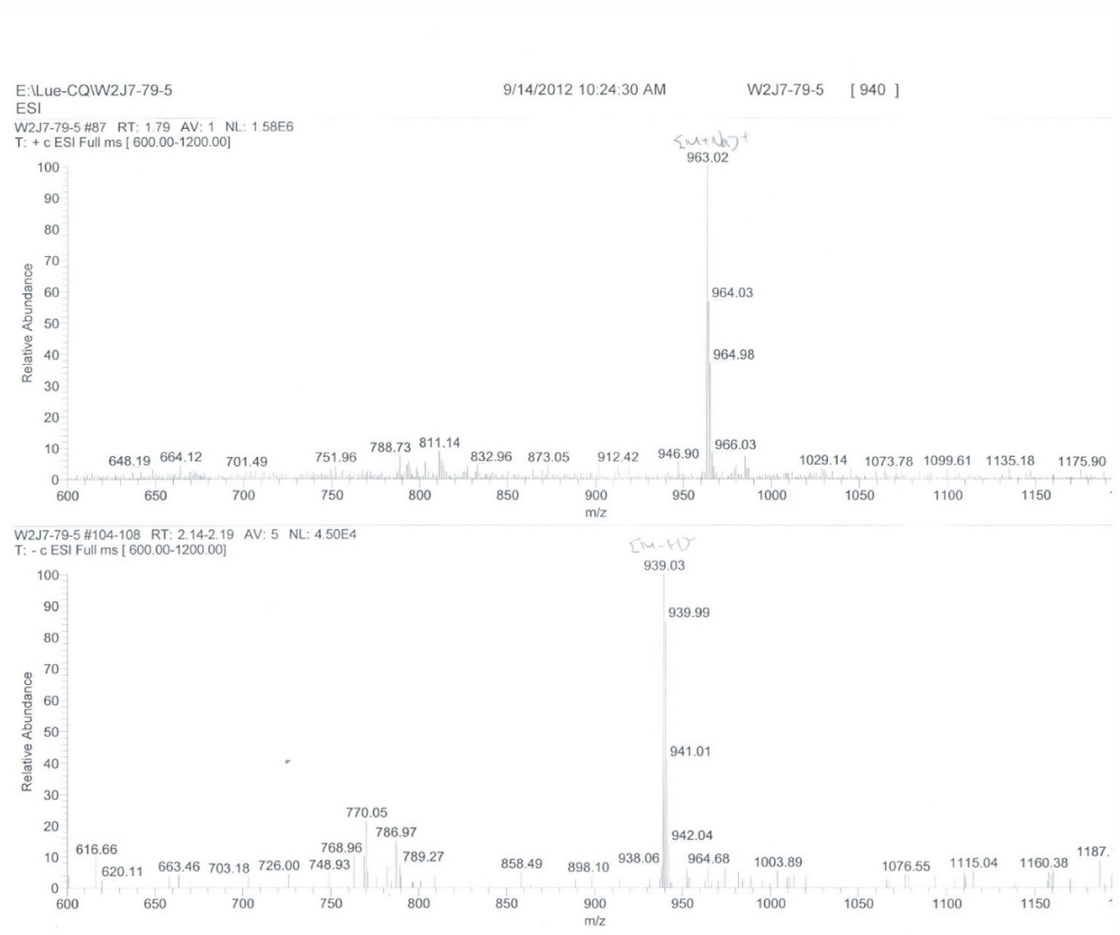

(**c**)

Figure S2. *Cont.*

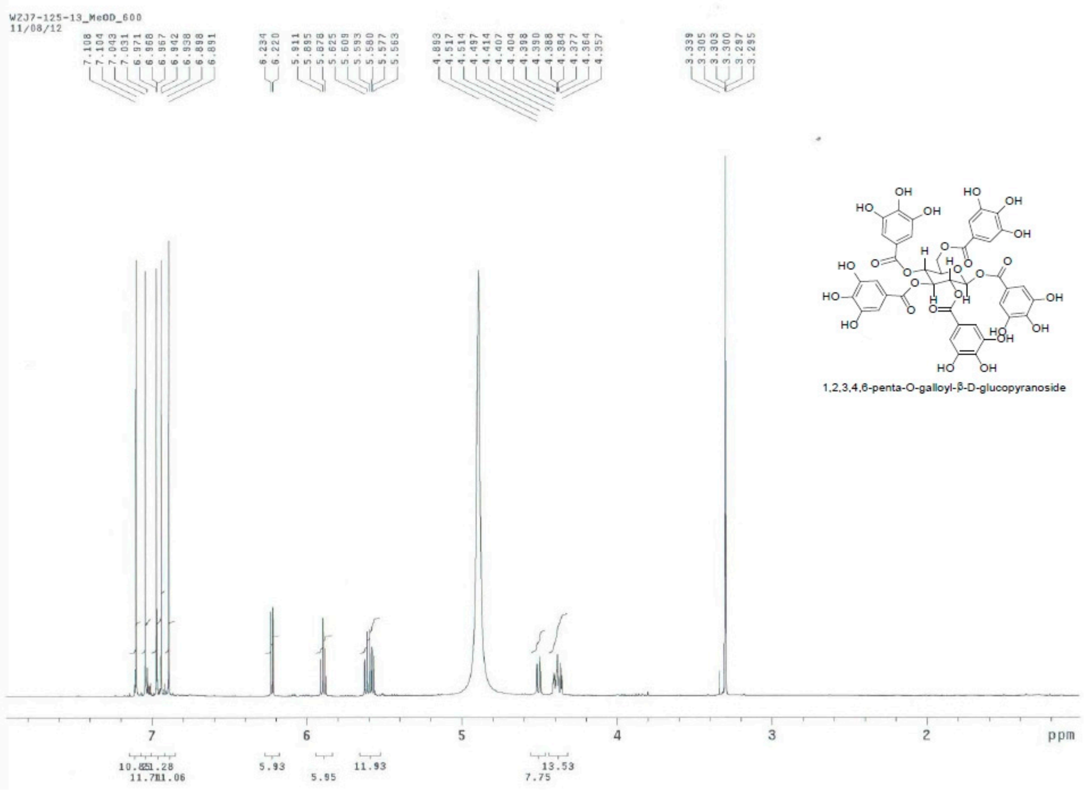

(**d**)

Figure S2. *Cont.*

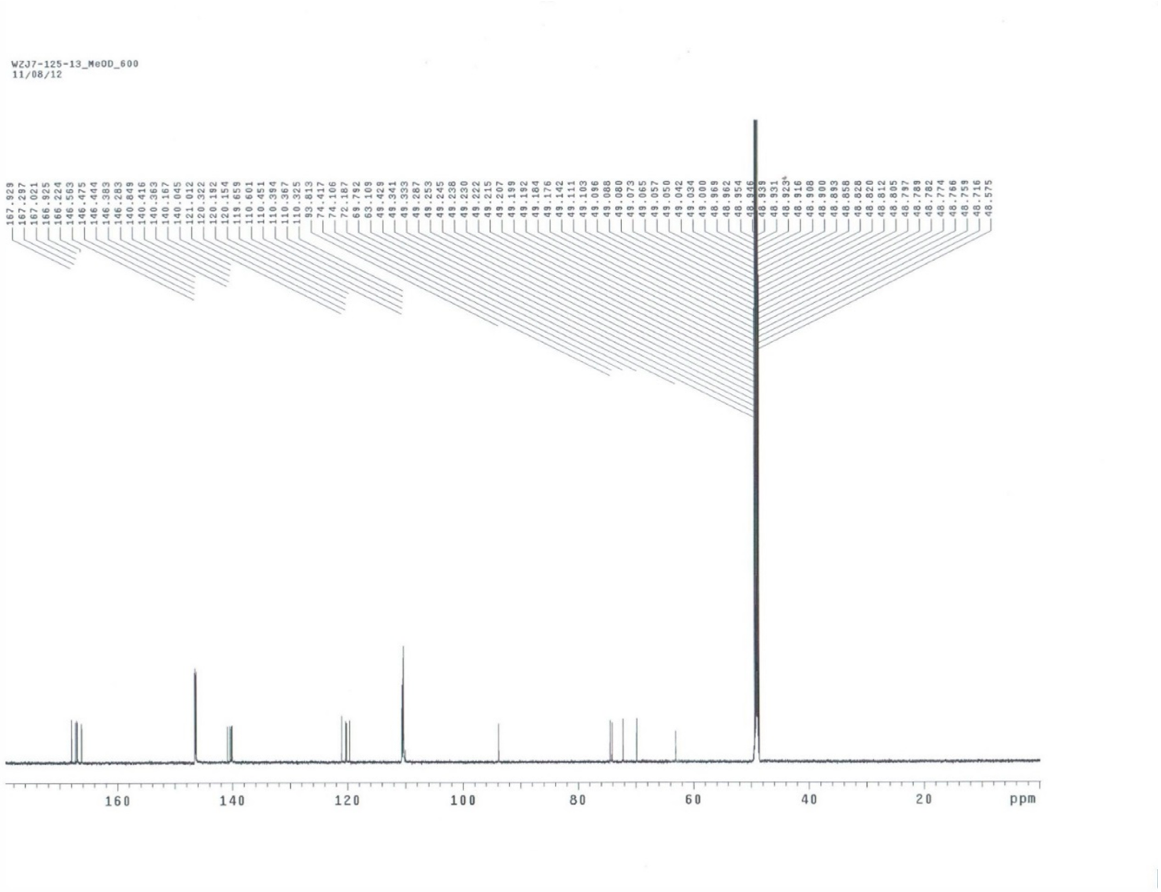

(**e**)

Figure S2. Chromatographs (**a**,**b**), mass spectrum (**c**) and nuclear magnetic resonance profiles (**d**,**e**) of 1,2,3,4,6-penta-*O*-galloyl-β-D-glucopyranoside (PGG). (**a**,**b**) The UPLC chromatographs of the bioactive fraction of F3-6 (**a**) and 1,2,3,4,6-penta-*O*-galloyl-β-D-glucopyranoside (PGG) (**b**). The analyses were performed on a Waters Acquity UPLC system including binary solvent manager, sampler manager, column compartment and Waters Photodiode Array Detector. The chromatographic data were collected and analyzed by Empower 2.0 software. The Thermo Syncronis C18 column (100 × 2.1 mm, 1.7 μm) was used. The column and sample temperature were maintained at 40 and 25 °C, respectively. The mobile phase consisted of water (0.1% phosphate buffer) (**a**) and acetonitrile (**b**) at a flow rate of 0.4 mL/min. The linear gradient was applied as following: 2% A, hold for 1 min; 2%–10% A, 1–2 min and hold for 2 min; 10%–25% A, 4–10 min; 25%–50% A, 10–14 min. The separation was followed by a 3-min washing procedure. The injection volume was 1 μL. The detection wavelength was set between 200 and 400 nm, and the chromatographs were extracted at 210 nm for purity check. 1,2,3,4,6-*O*-Penta-*O*-galloyl-β-D-glucopyranoside (PGG): a pale brown, amorphous powder; UV λ_{max} at 211, 231 and 278 nm; (**c**) ESI-MS (positive and negative ion mode) *m*/*z* 963.02 $[M + Na]^+$ and 939.03 [M − H]; (**d**) ^{1}H-NMR (CD_3OD) glucose moiety: δ 6.23 (d, 8.3 Hz, H-1), 5.89 (*t*, 9.6 Hz, H-3), 5.61 (*t*, 9.6 Hz, H-4), 5.58 (dd, 9.6, 8.3 Hz, H-2), 4.40 (m, H-5), 4.51 (d, 12.2 Hz, H-6a), 4.37 (dd, 12.2, 4.2 Hz, H6b). Galloyl moieties: δ 7.10, 7.04, 6.97, 6.94, 6.89 (s, each 2H); (**e**) ^{13}C-NMR (CD_3OD) glucose moiety: 93.9 (C-1), 74.3 (C-5), 74.1 (C-3), 72.1 (C-2), 69.8 (C-4), 63.1 (C-6). Galloyl moieties: 167.9, 167.3, 167.0, 166.9, 166.2 (carbonyl group signals), 146.6, 146.5, 146.4, 146.4, 146.3 (C-3, C-5), 140.9, 140.5, 140.4, 140.2, 140.1 (C-4), 121.0, 120.3, 120.2, 120.1, 119.6 (C-1), 110.6, 110.44, 110.38, 110.36, 110.3 (C-2, C-6).

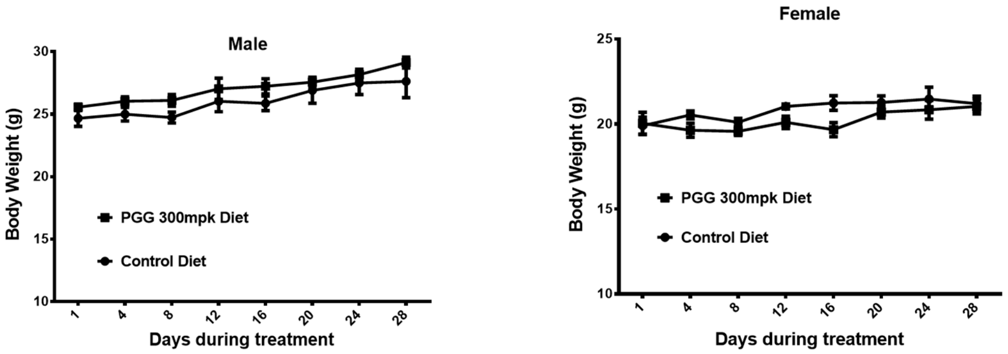

(**a**)

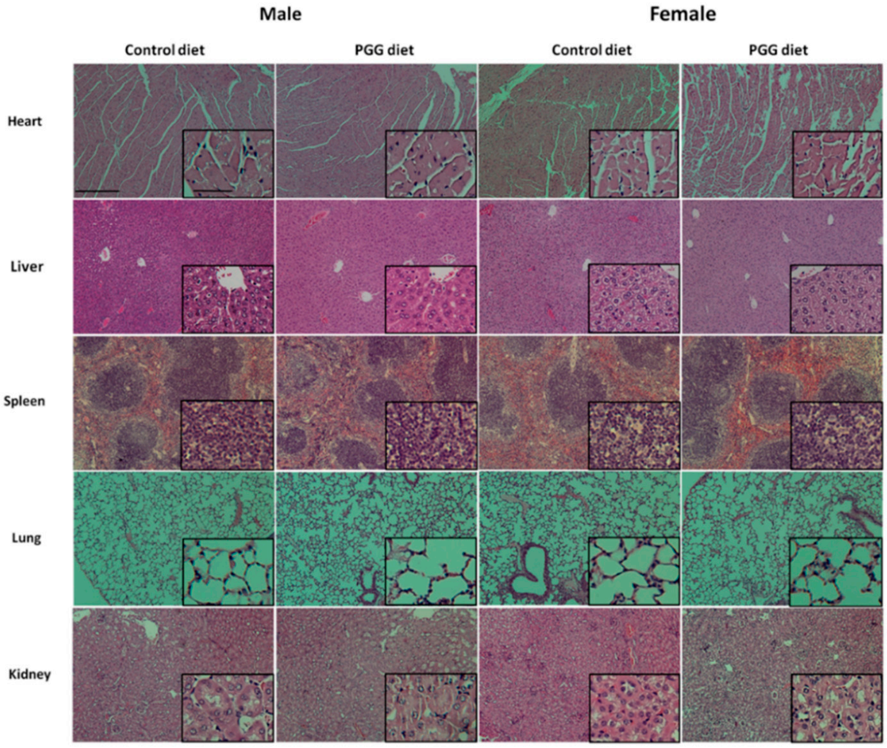

(**b**)

Figure S3. Safety of the dietary supplementation of PGG on mice. (**a**) Change of body weight of C57BL/6 mice ($n = 3$) fed with PGG mixed diet or control diet for 28 days. The error bars represent the standard deviations of three mice per group; (**b**) Representative H & E stained images of heart, liver, spleen, lung and kidney. Scale bars: 250 μm (original magnification, 100×) and 50 μm for insets (original magnification 200×). No evident changes was observed in the organs of the control diet group and the PGG mixed diet group.

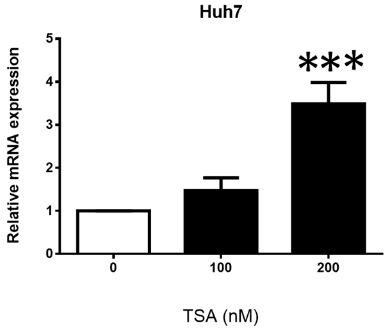

Figure S4. Effect of HDAC inhibitor on GNMT mRNA expression. Huh7 cells treated with TSA for 24 h. The GNMT mRNA level was determined by qRT-PCR. Data are expressed as the fold to solvent control. The graph shows the means ± SD ($n = 3$). *** $p < 0.01$. TSA = trichostatin A.

2. Supplementary Tables

Table S1. The list of drugs used for initial characterization of the assay platform.

No.	Drug Name	Catalog No. [a]	Fold Change (10 μM)
1	tanespimycin (geldanamycin)	A8476	0.5
2	camptothecin	C9911	0.4
3	pyrvinium	P0027	0.5
4	sanguinarine	S5890	0.1
5	withaferin A	W4394	0
6	mefloquine	M2319	0.3
7	mebendazole	M2523	1
8	chlorpromazine	C8138	0.7
9	sulconazole	S9632	0.5
10	bepridil	B5016	0.7
11	ciclopirox	C0415	8.2
12	clioquinol (quinolinol)	24880	3.8
13	GW-8510	G7791	0.4
14	prochlorperazine	P9178	0.7
15	thioridazine	T9025	0.1
16	tyloxapol	T0307	0.6
17	apigenin	10798	1.8
18	cloperastine	C2040	1
19	dipyridamole	D9766	0.9
20	luteolin	L9283	1.9
21	phenoxybenzamine	B019	0.7
22	DO 897/99	B9308	0.9
23	trifluoperazine	T8516	0.1
24	trioxysalen	T6137	1.2
25	suberoylanilide hydroxamic acid	S1047	18.6
26	trichostatin-A	T8552	3.4 [b]
27	sorafenib	S8599	0.9

[a] All drugs purchased from Sigma, except SAHA from Selleckchem.com and sorafenib from LC Laboratories; [b] concentration (0.2 μM).

Table S2. Ames pretest.

Metabolic Activation System	Dose (μg/plate)	Revertants Per Plate
		TA100
S9 mix (−)	Solvent control	69
	5000	22 *
	1000	23 *
	333.3	45
	100	67
	33.3	73
	10	63
	3.3	69
	Positive control	590
S9 mix (+)	Solvent control	75
	5000	28 *
	1000	40 *
	333.3	59
	100	65
	33.3	66
	10	60
	3.3	67
	Positive control	1388

Sterility Test	Colony Count
Test compound	0
S9 mix	0
Result	Sterile

* Pinpoint non-revertant colonies were observed.

Table S3. Ames result without metabolic activation (S9−).

**Metabolic Activation System**	**Dose (µg/plate)**	**Revertants Per Plate Mean ± SD**														
		**TA98**			**TA100**			**TA102**			**TA1535**			**TA1537**		
S9 mix (−)	Solvent control	7	7	12	61	32	52	177	185	115	5	10	10	4	5	4
		9 ± 2			48 ± 12			159 ± 31			8 ± 2			4 ± 0		
	333.3	9	12	9	46	40	43	103	91	167	6	14	6	6	3	7
		10 ± 1			43 ± 2			120 ± 33			9 ± 4			5 ± 2		
	100	12	10	19	44	46	79	151	123	159	7	11	8	5	9	2
		14 ± 4			56 ± 16			144 ± 15			9 ± 2			5 ± 3		
	33.3	9	20	12	60	54	63	135	172	149	10	9	10	2	8	5
		14 ± 5			59 ± 4			152 ± 15			10 ± 0			5 ± 2		
	10	7	9	13	57	54	63	156	108	178	7	10	4	11	4	8
		10 ± 2			58 ± 4			147 ± 29			7 ± 2			8 ± 3		
	3.3	15	12	16	76	67	69	175	160	161	6	12	13	5	5	10
		14 ± 2			71 ± 4			165 ± 7			10 ± 3			7 ± 2		
	Positive control	311	341	315	587	609	461	503	552	532	342	383	355	61	35	58
		322 ± 13			552 ± 65			529 ± 20			360 ± 17			51 ± 12		

**Sterility Test**	**Colony Count**
Test compound	0
Result	Sterile

Table S4. Ames result with metabolic activation (S9+).

Metabolic Activation System	Dose (μg/plate)	Revertants Per Plate Mean ± SD														
		TA98			TA100			TA102			TA1535			TA1537		
S9 mix (+)	Solvent control	14	11	18	72	30	27	244	245	103	7	7	13	19	11	15
		14 ± 3			43 ± 21			197 ± 67			9 ± 3			15 ± 3		
	333.3	11	15	17	49	65	54	100	99	103	5	6	7	6	9	11
		14 ± 2			56 ± 7			101 ± 2			6 ± 1			9 ± 2		
	100	17	21	27	48	59	60	99	138	121	5	6	9	10	15	11
		22 ± 4			56 ± 5			119 ± 16			7 ± 2			12 ± 2		
	33.3	30	28	11	61	52	50	141	150	149	14	8	9	9	6	13
		23 ± 9			54 ± 5			147 ± 4			10 ± 3			9 ± 3		
	10	25	11	19	40	72	58	172	141	155	11	8	4	18	13	20
		18 ± 6			57 ± 13			156 ± 13			8 ± 3			17 ± 3		
	3.3	18	22	26	83	65	54	145	151	132	6	14	11	10	15	12
		22 ± 3			67 ± 12			143 ± 8			10 ± 3			12 ± 2		
	Positive control	873	854	821	1124	1673	1813	965	1021	930	176	226	302	582	761	892
		849 ± 21			1537 ± 297			972 ± 37			235 ± 52			745 ± 127		

Sterility Test	Colony Count
Test compound	0
S9 mix	0
Result	Sterile

3. Supplementary Materials and Methods

Drug screening, extraction, isolation and identification of GNMT enhancer compounds, safety test in mice, diet and the Ames test:

3.1. Drug Screening

The traditional Chinese medicine library that contained 324 pure compounds (dissolved in DMSO at a concentration of 20 mg/mL) and 480 crude extracts (in DMSO at a concentration of 200 mg/mL) was provided from National Research Institute of Chinese Medicine. For primary screening, H7GPL cells seeded in 96-well plates were treated for 24 h with individual drugs at a concentration of 2 mg/mL for crude extracts and 0.2 mg/mL for pure compounds and then were lysed for the luciferase activity assay using the Luciferase Assay System (Promega, Madison, WI, USA). In each plate, six solvent control wells were treated with DMSO (the final concentration was 1%). Reporter activity for each well was transformed to the *Z* score by using data from all assay plates [35]. Then, hits of primary screening were sorted by Z score ≥1.5 and used for secondary screening. The same platform was used for secondary screening. Cells were treated with hits of the primary screen in duplicated plates for 20 h, and then alamarBlue® reagent (AbD serotec, Oxford, UK) was added into assay plates and incubated for four additional hours. The cytotoxicity was measured according to the manufacturer's recommendation, then was used to normalize the reporter activity and presented as the relative luciferase activity fold to control. Drugs that induced GNMT promoter activity ≥1.5-fold were considered as hits of the secondary screen.

3.2. Extraction, Isolation and Identification of GNMT Enhancer Compounds

Paeoniae radix rubra was purchased from a local Chinese drug store (Taipei, Taiwan) in May 2012. It was identified as the roots of *Paeonia lactiflora* Pall. About 150 g of the ground material was reflux extracted twice with 0.6 L of 50% aqueous MeOH for 1 h each. The supernatant was filtered through a filter paper, combined and partitioned three times with 0.7 L ethyl acetate each. The initial fractionation of the ethyl acetate extract was conducted by using an MPLC system (300 × 30 mm, silica gel, 40–63 μm; Merck, Germany). Dichloromethane (A) and MeOH (B) were used as the mobile phase (gradient conditions: 100% A for 1 h, to 40% B in 20 min and then to 100% B in 20 min, flow rate: 18 mL/min). The collected fractions were assayed by TLC (silica gel 60 F254 plates; Merck, Germany), using a mixture of ethyl acetate, MeOH and 0.1% acetic acid (15:2:0.5) as the mobile phase. Results were evaluated at 254 nm and by spraying with vanillin/sulfuric acid reagent. The luciferase assay was used to identify the active fractions in PL extract as described in the drug screening. Further purification of the most active fraction (F3) was performed on a Sephadex LH-20 column using MeOH as the mobile phase and obtained the bioactive fraction of F3-6.

The fraction F3-6 was further purified by a HPLC system, Agilent 1100 series coupled with a photodiode array detector. An RP-18 column (Cosmosil, 250 × 10 mm, 5 μm; nacalai, Japan) was used. Water (A) and CH_3CN (B), with 0.1% acetic acid each, were used as the mobile phase (gradient conditions: 5% B for 20 min, to 100% B, flow rate: 3 mL/min). Monitoring the separation at 203 nm led to the isolation of the effective compound, 1,2,3,4,6-penta-*O*-galloyl-β-D-glucopyranoside (PGG). PGG was obtained as a pale brown, amorphous powder with UV maxima at 211, 231 and 278 nm. For MS analysis, PGG was diluted in MeOH and directly infused into a Finnigan MATLCQ. The mass spectra were recorded in the positive and negative ESI mode and showed an m/z value of 963.02 $[M + Na]^+$ and 939.03 $[M - H]^-$, respectively. NMR spectra of the isolated compound in deuterated methanol (CD3OD) were recorded on a VNMRS 600 NMR spectrometer (Varian, Palo Alto, CA, USA). Identification was achieved by the comparison of the spectroscopic data obtained with those in the literature [36,37]. We used a 0.1-mg/mL concentration of purified fractions, F3-6 and PGG for most of the experiments, unless otherwise mentioned. PGG was dissolved in PBS for the cell-based assay.

3.3. Safety Test in Mice

Six-week-old male and female C57BL/6 mice (3 in each group) were purchased from BioLASCO (Taiwan) and acclimated in air-conditioned quarters at a room temperature of 20 ± 2 °C, relative humidity of 50% ± 10%, under 12:12 h light/dark conditions for 1 week. After a one-week acclimation period, mice were randomized in to the control (American Institute of Nutrition rodent (AIN93M) diet) and 0.25% PGG (*w*/*w* in AIN93M) diet. Body weight and food intake was assessed weekly. All mice were sacrificed by CO2 asphyxiation after 28 days of feeding, and organ samples were collected and stained with hematoxylin and eosin (H & E) for histopathological analysis. All animal experiments were reviewed and approved by the Institutional Animal Care and Use Committee of Kaohsiung Medical University.

3.4. Diet

PGG was purified from Paeonia lactiflora Pall and formulated into AIN93M (the American Institute of Nutrition rodent diet) basal diet at 0.25% *w*/*w*, to achieve an approximate daily dosing of 300 mg PGG/kg body weight, by Research Diets, Inc. (New Brunswick, NJ, USA) and stored at 4 °C in sealed bags.

3.5. Ames Test

Standard plate incorporation assays, with and without S9 activation, were conducted following the Organization for Economic Cooperation and Development (OECD) Test Number 471 recommendation (ref: Ames protocol; OECD). Briefly, the tester strains Salmonella typhimurium TA98, TA100, TA1535, TA1537 and TA102 were obtained from Moltox. One hundred microliters of PGG, dissolved in DMSO, were mixed with 100 μL of the tester strains (≥3 × 108 CFU/mL) and 2 mL of molten top agar before pouring onto minimal glucose agar plates. For the assay with S9 activation, an additional 500 μL of 5% S9 mix were added. Five logarithmic-diluted concentrations from 333 μg/plate, the highest concentration without bacterial toxicity, were tested in triplicate with and without S9 activation, respectively. The positive controls for the tester strains were 2-nitrofluorene (TA98; 0.5 μg/plate), sodium azide (TA100 and TA1535; 1.5 μg/plate), 9-aminoacridine (TA1537; 50 μg/plate) and mitomycin C (TA102; 5 μg/plate) in the absence of S9 activation. For all tester strains with S9 activation, 2-aminoanthracene (10, 4, 10, 8 and 10 μg/plate for TA98, TA100, TA1535, TA1537 and TA102, respectively) was used as the positive control [38,39].
